# Poly[tetra­butyl­ammonium [chlorido­hexa­methyl-μ_3_-sulfato-distannate(IV)]]

**DOI:** 10.1107/S1600536813016723

**Published:** 2013-06-22

**Authors:** Tidiane Diop, Arie van der Lee, Mamadou Sidibé

**Affiliations:** a Laboratoire de Chimie Minérale et Analytique, Département de Chimie, Faculté des Sciences et Techniques, Université Cheikh Anta Diop, Dakar, Senegal; b Institut Européen des Membranes, Université de Montpellier II, 34000 Montpellier, France

## Abstract

In the structure of the title coordination polymer, {(C_16_H_36_N)[Sn_2_(CH_3_)_6_Cl(SO_4_)]}_*n*_, the two independent Sn^IV^ atoms are coordinated in a trigonal–bipyramidal manner by three methyl groups in the equatorial plane and in the axial positions by either two O atoms of bridging SO_4_^2−^ anions or by a Cl atom and one O atom of a bridging SO_4_^2−^ anion, respectively. The [Sn_2_(CH_3_)_6_Cl(SO_4_)]^−^ anion forms an infinite zigzag chain parallel to the *c* axis. The cations are situated between these chains. Two of the four butyl groups of the cation are partially disordered over two sets of sites with site occupancies of 0.79 (2):0.21 (2) and 0.75 (2):0.25 (2), respectively. Weak C—H⋯O hydrogen-bonding inter­actions help to consolidate the crystal packing.

## Related literature

For related structures, see: Molloy *et al.* (1989 ▶); Zhang *et al.* (2008 ▶); Sadiq-ur-Rehman *et al.* (2004 ▶); Aziz-ur-Rehman *et al.* (2006 ▶); Diallo *et al.* (2009 ▶); Diop *et al.* (2012 ▶). For details of the use of constraints and restraints during the structure refinement, see: Cooper *et al.* (2010 ▶, 2012 ▶). For background to the weighting schemes used in the refinement, see: Prince (1982 ▶); Watkin (1994 ▶).
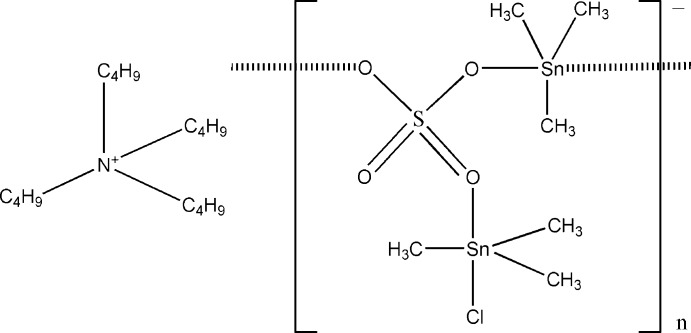


## Experimental

### 

#### Crystal data

(C_16_H_36_N)[Sn_2_(CH_3_)_6_Cl(SO_4_)]
*M* *_r_* = 701.60
Orthorhombic, 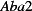

*a* = 27.2051 (6) Å
*b* = 20.4336 (5) Å
*c* = 11.4370 (2) Å
*V* = 6357.8 (3) Å^3^
*Z* = 8
Mo *K*α radiation
μ = 1.75 mm^−1^
*T* = 175 K
0.25 × 0.20 × 0.15 mm

#### Data collection

Agilent Xcalibur (Sapphire3, Gemini) diffractometer
Absorption correction: multi-scan (*CrysAlis PRO*; Agilent, 2010 ▶) *T*_min_ = 0.651, *T*_max_ = 1.000
59083 measured reflections
8068 independent reflections
7179 reflections with *I* > 2.0σ(*I*)
*R*_int_ = 0.049

#### Refinement

*R*[*F* ^2^ > 2σ(*F* ^2^)] = 0.053
*wR*(*F* ^2^) = 0.053
*S* = 1.07
7179 reflections
301 parameters
33 restraints
H-atom parameters constrained
Δρ_max_ = 1.44 e Å^−3^
Δρ_min_ = −2.06 e Å^−3^
Absolute structure: Flack (1983 ▶), 3709 Friedel pairs
Flack parameter: 0.05 (4)

### 

Data collection: *CrysAlis PRO* (Agilent, 2010 ▶); cell refinement: *CrysAlis PRO*; data reduction: *CrysAlis PRO*; program(s) used to solve structure: *SUPERFLIP* (Palatinus & Chapuis, 2007 ▶); program(s) used to refine structure: *CRYSTALS* (Betteridge *et al.*, 2003 ▶); molecular graphics: *OLEX2* (Dolomanov *et al.*, 2009 ▶); software used to prepare material for publication: *CRYSTALS*.

## Supplementary Material

Crystal structure: contains datablock(s) global, I. DOI: 10.1107/S1600536813016723/wm2750sup1.cif

Structure factors: contains datablock(s) I. DOI: 10.1107/S1600536813016723/wm2750Isup2.hkl

Additional supplementary materials:  crystallographic information; 3D view; checkCIF report

## Figures and Tables

**Table 1:** Selected bond lengths (Å)

Sn1—C3	2.112 (7)
Sn1—C4	2.124 (7)
Sn1—C5	2.117 (7)
Sn1—Cl2	2.5561 (18)
Sn1—O6	2.345 (4)
Sn11—C12	2.090 (7)
Sn11—C13	2.108 (6)
Sn11—C14	2.088 (7)
Sn11—O9^i^	2.269 (5)
Sn11—O10	2.286 (5)

Symmetry code: (i) 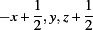.

**Table 2:** Hydrogen-bond geometry (Å, °)

*D*—H⋯*A*	*D*—H	H⋯*A*	*D*⋯*A*	*D*—H⋯*A*
C16—H161⋯O8^ii^	0.98	2.50	3.386 (18)	150 (1)
C28—H281⋯O8^ii^	0.97	2.43	3.195 (18)	136 (1)
C25—H251⋯O8^ii^	0.95	2.48	3.434 (18)	175 (1)

Symmetry code: (ii) 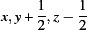.

**Fig. 1.**
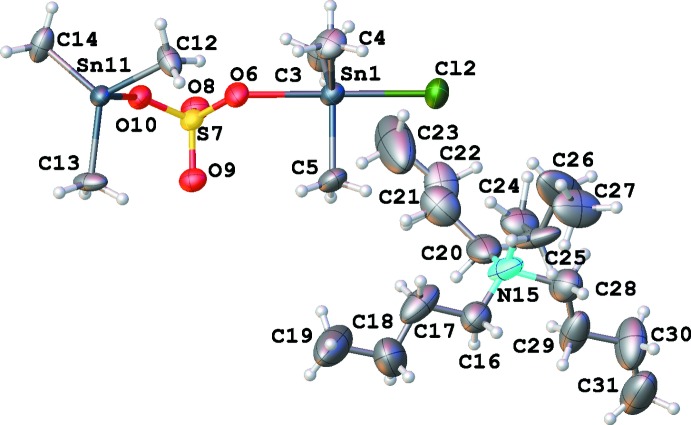
A view of the asymmetric unit of the title compound, showing the numbering scheme and displacement ellipsoids drawn at the 50% probability level. For the disordered part of the cation, only the mojor component is displayed. H atoms are shown as spheres of arbitary radius.

**Fig. 2.**
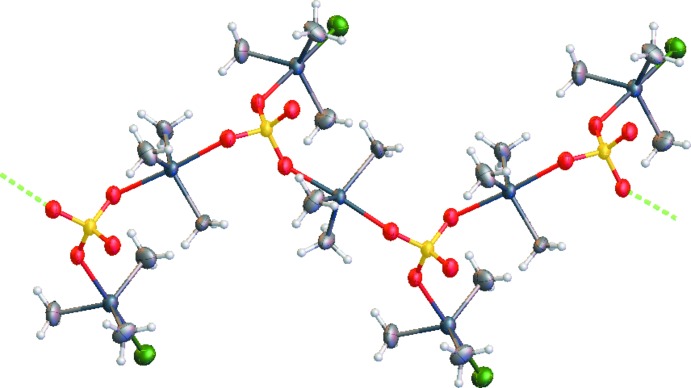
A view of the polymeric chain of the organotin(IV) complex anion, [Sn2(CH3)6Cl(SO4)]-; displacement ellipsoids are drawn at the 50% probability level.

**Fig. 3.**
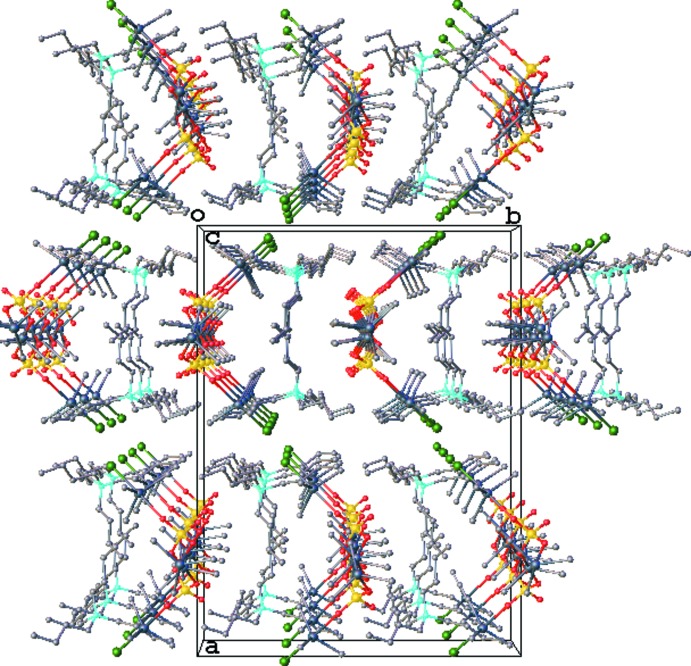
A view of the packing of the title compound. Hydrogen atoms have been omitted for clarity.

## supplementary crystallographic information

### Comment

Among organotin(IV) complexes, a number of trimethyltin derivatives form polymeric structures with trigonal bipyramidal geometry around the Sn^IV^ atom, e.g. as reported by Molloy *et al.* (1989); Sadiq-ur-Rehman *et al.* (2004); Aziz-ur-Rehman *et al.* (2006); Zhang *et al.* (2008).

The title compound, {(C_16_H_36_N)[Sn_2_(CH_3_)_6_Cl(SO_4_)]}_n_, crystallizes with one tetrabutylammonium cation, Bu_4_N^+^, and one organotin(IV) complex anion in the asymmetric unit (Fig. 1). The two independent Sn^IV^ atoms within the *trans*-O*X*SnC_3_ (*X* = O, Cl) moieties have a distorted trigonal-bipyramidal environment. The axial positions involve either two O atoms of different sulfate anions [Sn11], or one O atom of a sulfate anion and a Cl atom [Sn1]; both Sn^IV^ atoms are bonded to three methyl groups in equatorial positions. The axial angle O10—Sn11—O9 is 176.23 (15)° and is more distorted from the ideal angle of linearity compared to Cl2—Sn1—O6 (177.89 (12)°). The axial Sn—O distances (Sn1—O6 2.345 (4) Å; Sn11—O10 2.286 (5) Å; Sn11—O9 2.269 (5) Å; Table 1) are shorter than the Sn—O distance of 2.450 (5) Å in the structure of the related compound (Bu_4_N)[Sn(CH_3_)_3_Cl(HSO_4_)] (Diallo *et al.*, 2009), but are in the excepted range [2.262 (2)–2.305 (2) Å] found in (Bu_4_N)[Sn_3_(CH_3_)_9_(SO_4_)_2_] (Diop *et al.*, 2012).

The [Sn_2_(CH_3_)_6_Cl(SO_4_)]^-^ anionic units in the title compound assemble into an infinite zigzag chain parallet to the *c*-axis formed by (SnMe_3_) units bridged by SO_4_^2-^ units (Fig. 2). The SO_4_^2-^ µ_3_-bridging anion itself bonds to a third (SnMe_3_)Cl unit. The [Sn_2_(CH_3_)_6_Cl(SO_4_)]^-^_∞_ chains are separated by Bu_4_N^+^ cations as to form distinct layers parallel to the *bc* plane (Fig. 3). Weak C—H···O hydrogen bond interactions (Table 2) are present between the sulfate O8 atoms and butyl chains of the Bu_4_N^+^ cations.

### Experimental

Ethanolic solutions containing (Bu_4_N)HSO_4_ (1.26 g, 4 mmol) and SnMe_3_Cl (1.59 g, 8 mmol) were mixed and stirred at room temperature for more than 1 h. After removing the precipitate, the filtrate was allowed to evaporate to yield colourless crystals of the title compound. The overall reaction is: (Bu_4_N)HSO_4_ + 2SnMe_3_Cl → (Bu_4_N)[Sn_2_(CH_3_)_6_Cl(SO_4_)] + HCl

### Refinement

Most of the H atoms were located in a difference map, but they were repositioned geometrically. The H atoms were initially refined with soft restraints on the bond lengths and angles to regularize their geometry (C—H in the range 0.93–0.98, Å) and *U*_iso_(H) (in the range 1.2–1.5 times *U*_eq_ of the parent atom), after which the positions were refined with riding constraints (Cooper *et al.*, 2010). The severe disorder in two of the four butyl chains of the tetrabutylammonium cation was treated by introducing a new carbon site and refining the occupancies of the two sets of sites. Restraints on the interatomic distances and on the anisotropic atomic displacement parameters were necessary in order to obtain a reasonable geometry. Initially shift limiting restraints were applied but in the final stages of the refinement removed. It proved also be necessary to apply interatomic distance restraints between pairs of non-splitted carbon atoms. Asymmetric atomic displacement restraints were used for the atomic displacement parameters of the disordered carbon atoms (Cooper *et al.*, 2012). The size and shape of other carbon atoms in the butyl chains suggest that they are most probably disordered as well, but the disorder does not appear to be well-resolved, and it could not be modelled satisfactorily. The tree reflections (0 2 0), (1 1 1) and (2 0 0) have been omitted from the refinement, because they were found to be partially masked by the beamstop. The highest positive difference electron density, 1.44 e^-^ Å^-3^, is found at 0.42 Å from C20, whereas the largest negative electron density, -2.06 e^-^ Å^-3^, is found at 0.83 Å from C14.

### Crystal data

**Table:**

(C_16_H_36_N)[Sn_2_(CH_3_)_6_Cl(SO_4_)]	*F*(000) = 2864
*M**_r_* = 701.60	*D*_x_ = 1.466 Mg m^−^^3^
Orthorhombic, *A**b**a*2	Mo *K*α radiation, λ = 0.71073 Å
Hall symbol: A 2 -2ac	Cell parameters from 17152 reflections
*a* = 27.2051 (6) Å	θ = 2.0–27.7°
*b* = 20.4336 (5) Å	µ = 1.75 mm^−^^1^
*c* = 11.4370 (2) Å	*T* = 175 K
*V* = 6357.8 (3) Å^3^	Prism, colourless
*Z* = 8	0.25 × 0.20 × 0.15 mm

### Data collection

**Table:**

Agilent Xcalibur (Sapphire3, Gemini) diffractometer	8068 independent reflections
Radiation source: Enhance (Mo) X-ray Source	7179 reflections with *I* > 2.0σ(*I*)
Graphite monochromator	*R*_int_ = 0.049
Detector resolution: 16.0143 pixels mm^-1^	θ_max_ = 29.4°, θ_min_ = 1.5°
ω scans	*h* = −37→34
Absorption correction: multi-scan (*CrysAlis PRO*; Agilent, 2010)	*k* = −27→26
*T*_min_ = 0.651, *T*_max_ = 1.000	*l* = −15→15
59083 measured reflections	

### Refinement

**Table:**

Refinement on *F*	Hydrogen site location: difference Fourier map
Least-squares matrix: full	H-atom parameters constrained
*R*[*F*^2^ > 2σ(*F*^2^)] = 0.053	Method, part 1, Chebychev polynomial, (Watkin, 1994, *P*rince, 1982) [*w*eight] = 1.0/[A_0_*T_0_(x) + A_1_*T_1_(x) ··· + A_n-1_]*T_n-1_(x)] where A_i_ are the Chebychev coefficients listed belo*w* and x = *F* /*F*max Method = Robust Weighting (*P*rince, 1982) W = [*w*eight] * [1-(delta*F*/6*sigma*F*)^2^]^2^ A_i_ are: 13.1 -3.14 6.69 3.90 -2.02
*wR*(*F*^2^) = 0.053	(Δ/σ)_max_ = 0.001
*S* = 1.07	Δρ_max_ = 1.44 e Å^−^^3^
7179 reflections	Δρ_min_ = −2.06 e Å^−^^3^
301 parameters	Absolute structure: Flack (1983), 3709 Friedel pairs
33 restraints	Flack parameter: 0.05 (4)
Primary atom site location: iterative	

### Special details

**Table:**

Refinement. Friedif = 325.1 Estimated Friedel difference = 106.9462 f computed from scattering factors, including f-primeCurrent Do—Dc *R*-factor (%)= 79.99No of Reflections processed = 7181 No of Friedel Pairs found = 3241 No of Friedel Pairs used = 3241 No of Unpaired Reflections = 375 No of Centric Reflections = 324 Flack parameter obtained from original refinement Hooft parameter obtained with Flack *x* set to zero Reflections only used if /*F_o_*+ - *F_o_*-/ < 99999.00 * /Fc+ - Fc-/ Friedif = 325.12 Acta A63, (2007), 257–265 Flack & Shmueli (2007) recommend a value >200 for general structures and >80 for enantiopure crystalsFlack Parameter & su 0.0477 0.0352 Hooft Parameter & su 0.0130 0.0149 Ton G & su 0.9739 0.0299No of reflections for which delta(*F_o_*) has same sign as delta(Fc) Same sign Opposite sign 2050 1191For an enantiopure material, there are 2 choices, P2 P2(correct) 1.0000 *i.e.* 0.100000E+01If 50:50 twinning is possible, there are 3 choices, P3 P3(correct) 1.0000 *i.e.* 0.100000E+01 P3(rac-twin) 0.0000 *i.e.* 0.000000E+00 P3(inverse) 0.0000 *i.e.* 0.000000E+00 G 0.9739 G *S*.U. 0.0299 FLEQ 0.0130 FLEQ *S*.U. 0.0149 *i.e.* 0.149430E-01

### Fractional atomic coordinates and isotropic or equivalent isotropic displacement parameters (Å^2^)

**Table:**

	*x*	*y*	*z*	*U*_iso_*/*U*_eq_	Occ. (<1)
Sn1	0.095395 (14)	0.642868 (19)	0.30555 (17)	0.0371	
Cl2	0.03842 (7)	0.72492 (9)	0.2051 (2)	0.0553	
C3	0.0556 (3)	0.5604 (4)	0.2468 (8)	0.0574	
C4	0.0795 (3)	0.6783 (4)	0.4760 (6)	0.0567	
C5	0.1567 (3)	0.6826 (4)	0.2163 (8)	0.0594	
O6	0.14856 (17)	0.5707 (2)	0.4025 (4)	0.0407	
S7	0.18192 (5)	0.51883 (7)	0.3647 (2)	0.0326	
O8	0.15838 (16)	0.4724 (2)	0.2867 (4)	0.0428	
O9	0.22483 (15)	0.5494 (2)	0.3060 (4)	0.0395	
O10	0.19910 (16)	0.4841 (2)	0.4713 (4)	0.0398	
Sn11	0.239704 (13)	0.516838 (18)	0.63624 (17)	0.0326	
C12	0.1884 (3)	0.5899 (4)	0.6739 (6)	0.0512	
C13	0.3026 (2)	0.5367 (5)	0.5346 (6)	0.0503	
C14	0.2271 (3)	0.4218 (3)	0.6961 (7)	0.0549	
N15	0.1078 (2)	0.8008 (4)	−0.1571 (6)	0.0660	
C16	0.1614 (3)	0.8095 (5)	−0.1593 (8)	0.0694	
C17	0.1865 (4)	0.7718 (5)	−0.0667 (8)	0.0909	
C18	0.2411 (4)	0.7755 (8)	−0.0881 (16)	0.1209	
C19	0.2674 (8)	0.7363 (10)	0.0005 (16)	0.1779	
C20	0.0925 (5)	0.7338 (4)	−0.1953 (10)	0.0847	0.75 (2)
C21	0.0846 (4)	0.6772 (5)	−0.1147 (8)	0.0905	
C22	0.0556 (4)	0.6258 (4)	−0.1772 (8)	0.0845	
C23	0.0525 (8)	0.5731 (10)	−0.0870 (15)	0.2003	
C24	0.0770 (4)	0.8207 (5)	−0.0658 (9)	0.0760	
C25	0.0910 (4)	0.8850 (5)	−0.0131 (9)	0.0844	0.79 (2)
C26	0.0555 (5)	0.9121 (6)	0.0743 (15)	0.1389	
C27	0.0722 (5)	0.9755 (5)	0.1224 (15)	0.1060	
C28	0.0855 (4)	0.8509 (6)	−0.2424 (8)	0.0762	
C29	0.1037 (4)	0.8465 (5)	−0.3628 (8)	0.0891	
C30	0.0773 (7)	0.8833 (9)	−0.4543 (9)	0.1188	
C31	0.0985 (6)	0.8812 (9)	−0.5722 (12)	0.1342	
C251	0.0409 (7)	0.8720 (12)	−0.029 (2)	0.0842	0.21 (2)
C201	0.0734 (7)	0.7488 (5)	−0.115 (3)	0.0825	0.25 (2)
H161	0.1685	0.8563	−0.1489	0.0840*	
H162	0.1733	0.7947	−0.2355	0.0843*	
H171	0.1783	0.7893	0.0092	0.1081*	
H172	0.1768	0.7268	−0.0721	0.1082*	
H231	0.0361	0.5360	−0.1206	0.2980*	
H232	0.0343	0.5883	−0.0207	0.2980*	
H233	0.0851	0.5606	−0.0634	0.2980*	
H271	0.0515	1.0102	0.0937	0.1599*	
H272	0.1053	0.9833	0.0977	0.1599*	
H273	0.0712	0.9748	0.2063	0.1600*	
H41	0.1065	0.6699	0.5270	0.0864*	
H42	0.0731	0.7248	0.4738	0.0860*	
H43	0.0511	0.6560	0.5060	0.0862*	
H31	0.0631	0.5239	0.2949	0.0852*	
H32	0.0211	0.5696	0.2514	0.0851*	
H33	0.0645	0.5508	0.1666	0.0850*	
H51	0.1509	0.7279	0.2013	0.0890*	
H52	0.1607	0.6600	0.1437	0.0891*	
H53	0.1855	0.6773	0.2625	0.0892*	
H121	0.2056	0.6301	0.6864	0.0762*	
H122	0.1705	0.5780	0.7432	0.0765*	
H123	0.1664	0.5950	0.6084	0.0760*	
H141	0.2542	0.4064	0.7409	0.0820*	
H142	0.1979	0.4221	0.7426	0.0822*	
H143	0.2219	0.3935	0.6305	0.0822*	
H131	0.3044	0.5074	0.4683	0.0762*	
H132	0.3316	0.5311	0.5806	0.0760*	
H133	0.3011	0.5809	0.5077	0.0760*	
H221	0.0235	0.6429	−0.1972	0.1000*	
H222	0.0715	0.6107	−0.2481	0.0999*	
H301	0.0769	0.9290	−0.4317	0.1441*	
H302	0.0438	0.8664	−0.4578	0.1442*	
H291	0.1371	0.8637	−0.3623	0.1052*	
H292	0.1037	0.8006	−0.3855	0.1051*	
H281	0.0908	0.8950	−0.2139	0.0911*	
H282	0.0503	0.8419	−0.2460	0.0910*	
H181	0.2519	0.8205	−0.0842	0.1501*	
H182	0.2482	0.7580	−0.1652	0.1502*	
H251	0.1081	0.9092	−0.0717	0.1240*	0.79 (2)
H252	0.1136	0.8866	0.0508	0.1240*	0.79 (2)
H2511	0.0149	0.8854	−0.0808	0.1240*	0.21 (2)
H2512	0.0143	0.8465	0.0015	0.1240*	0.21 (2)
H201	0.0696	0.7337	−0.2582	0.1321*	0.75 (2)
H202	0.1261	0.7214	−0.2043	0.1319*	0.75 (2)
H2011	0.0744	0.7279	−0.0401	0.1290*	0.25 (2)
H2012	0.0381	0.7501	−0.1125	0.1290*	0.25 (2)
H241	0.0414	0.8104	−0.0162	0.1181*	0.79 (2)
H242	0.0819	0.8014	0.0099	0.1122*	0.79 (2)
H243	0.1035	0.8274	0.0073	0.1180*	0.21 (2)
H244	0.0764	0.7722	−0.0157	0.1205*	0.21 (2)
H211	0.1157	0.6638	−0.0821	0.1384*	0.75 (2)
H212	0.1170	0.6617	−0.0949	0.1383*	0.75 (2)
H213	0.1181	0.6647	−0.0991	0.1381*	0.25 (2)
H214	0.1169	0.6649	−0.0885	0.1381*	0.25 (2)
H311	0.0927	0.9220	−0.6108	0.2010*	
H312	0.1332	0.8731	−0.5670	0.2010*	
H313	0.0831	0.8467	−0.6159	0.2011*	
H191	0.2959	0.7172	−0.0350	0.2810*	
H192	0.2772	0.7643	0.0636	0.2810*	
H193	0.2461	0.7024	0.0289	0.2809*	
H261	0.0247	0.9184	0.0368	0.1704*	0.79 (2)
H262	0.0518	0.8817	0.1366	0.1704*	0.79 (2)
H263	0.0269	0.9062	0.1207	0.1704*	0.21 (2)
H264	0.0812	0.8851	0.1033	0.1704*	0.21 (2)

### Atomic displacement parameters (Å^2^)

**Table:**

	*U*^11^	*U*^22^	*U*^33^	*U*^12^	*U*^13^	*U*^23^
Sn1	0.04077 (18)	0.03904 (18)	0.03139 (16)	−0.00380 (16)	−0.00108 (18)	0.00268 (18)
Cl2	0.0568 (10)	0.0501 (9)	0.0589 (10)	0.0069 (8)	−0.0059 (8)	0.0132 (8)
C3	0.055 (4)	0.051 (4)	0.066 (5)	−0.010 (3)	−0.023 (4)	0.000 (3)
C4	0.077 (5)	0.065 (5)	0.028 (3)	0.001 (4)	0.000 (3)	−0.008 (3)
C5	0.056 (4)	0.062 (5)	0.060 (4)	−0.008 (4)	0.008 (4)	0.026 (4)
O6	0.046 (2)	0.051 (2)	0.0252 (19)	0.005 (2)	−0.0002 (17)	0.0042 (17)
S7	0.0359 (7)	0.0386 (7)	0.0232 (6)	−0.0024 (6)	−0.0004 (5)	0.0037 (5)
O8	0.046 (2)	0.050 (2)	0.032 (2)	−0.0083 (18)	−0.0009 (17)	−0.0023 (18)
O9	0.0439 (19)	0.047 (2)	0.0278 (17)	−0.0062 (17)	−0.0002 (19)	−0.002 (2)
O10	0.042 (2)	0.044 (2)	0.0330 (19)	0.0026 (18)	0.0010 (17)	0.0058 (19)
Sn11	0.03353 (15)	0.03812 (17)	0.02608 (14)	0.00369 (15)	0.00169 (16)	0.00763 (18)
C12	0.047 (4)	0.073 (5)	0.034 (3)	0.024 (3)	−0.006 (2)	−0.010 (3)
C13	0.038 (3)	0.087 (6)	0.026 (3)	−0.018 (3)	0.007 (2)	0.004 (3)
C14	0.075 (5)	0.036 (3)	0.053 (4)	−0.004 (3)	−0.023 (4)	0.007 (3)
N15	0.065 (4)	0.088 (5)	0.045 (3)	−0.027 (4)	0.002 (3)	0.023 (3)
C16	0.073 (5)	0.068 (5)	0.067 (5)	0.009 (4)	0.009 (4)	0.009 (4)
C17	0.165 (13)	0.059 (5)	0.048 (5)	−0.004 (6)	0.016 (6)	0.007 (4)
C18	0.089 (9)	0.116 (11)	0.158 (18)	0.032 (9)	0.018 (9)	0.041 (11)
C19	0.22 (2)	0.145 (16)	0.165 (19)	0.102 (16)	0.116 (18)	0.086 (15)
C20	0.077 (7)	0.126 (12)	0.051 (6)	0.019 (8)	0.022 (7)	0.020 (9)
C21	0.085 (8)	0.108 (9)	0.079 (7)	0.001 (6)	−0.011 (5)	−0.029 (7)
C22	0.109 (8)	0.089 (7)	0.055 (6)	0.029 (6)	−0.024 (5)	−0.039 (5)
C23	0.19 (2)	0.33 (3)	0.086 (11)	0.14 (2)	0.003 (12)	0.036 (16)
C24	0.082 (6)	0.082 (7)	0.063 (5)	0.004 (5)	0.021 (5)	−0.017 (5)
C25	0.082 (8)	0.128 (12)	0.044 (6)	−0.059 (8)	0.006 (5)	0.023 (7)
C26	0.088 (8)	0.052 (6)	0.28 (2)	−0.017 (5)	0.038 (12)	−0.018 (9)
C27	0.125 (10)	0.067 (6)	0.126 (11)	−0.009 (6)	0.045 (9)	−0.050 (7)
C28	0.073 (6)	0.094 (7)	0.061 (5)	−0.002 (5)	−0.006 (4)	0.011 (5)
C29	0.093 (7)	0.063 (5)	0.112 (9)	−0.022 (5)	−0.045 (7)	0.030 (6)
C30	0.160 (13)	0.131 (13)	0.066 (7)	0.059 (11)	−0.028 (8)	0.000 (7)
C31	0.112 (11)	0.129 (12)	0.161 (17)	−0.027 (10)	−0.037 (11)	0.076 (12)
C251	0.081 (4)	0.127 (5)	0.044 (5)	−0.059 (5)	0.009 (5)	0.025 (5)
C201	0.077 (5)	0.124 (5)	0.046 (4)	0.018 (5)	0.019 (4)	0.022 (5)

### Geometric parameters (Å, º)

**Table:**

Sn1—C3	2.112 (7)	C21—C22	1.4946 (10)
Sn1—C4	2.124 (7)	C21—C201	1.4949 (10)
Sn1—C5	2.117 (7)	C21—H211	0.963
Sn1—Cl2	2.5561 (18)	C21—H212	0.963
Sn1—O6	2.345 (4)	C21—H213	0.961
Sn11—C12	2.090 (7)	C21—H214	0.960
Sn11—C13	2.108 (6)	C22—C23	1.4951 (10)
Sn11—C14	2.088 (7)	C22—H221	0.967
Sn11—O9^i^	2.269 (5)	C22—H222	0.969
Sn11—O10	2.286 (5)	C23—H231	0.960
C3—H31	0.949	C23—H232	0.958
C3—H32	0.960	C23—H233	0.962
C3—H33	0.969	C24—C25	1.4949 (10)
C4—H41	0.955	C24—C251	1.4950 (10)
C4—H42	0.966	C24—C201	1.58 (2)
C4—H43	0.960	C24—H241	1.143
C5—H51	0.955	C24—H242	0.962
C5—H52	0.956	C24—H243	1.113
C5—H53	0.951	C24—H244	1.147
O6—S7	1.460 (5)	C25—C26	1.4953 (10)
S7—O8	1.451 (5)	C25—C251	1.40 (3)
S7—O9	1.485 (4)	C25—H251	0.954
S7—O10	1.487 (4)	C25—H252	0.956
C12—H121	0.957	C25—H243	1.247
C12—H122	0.961	C26—C27	1.479 (15)
C12—H123	0.964	C26—C251	1.4949 (10)
C13—H131	0.967	C26—H261	0.950
C13—H132	0.955	C26—H262	0.950
C13—H133	0.955	C26—H263	0.950
C14—H141	0.953	C26—H264	0.950
C14—H142	0.954	C27—H271	0.962
C14—H143	0.957	C27—H272	0.958
N15—C16	1.469 (8)	C27—H273	0.960
N15—C20	1.4958 (10)	C28—C29	1.466 (9)
N15—C24	1.399 (11)	C28—H281	0.968
N15—C28	1.539 (8)	C28—H282	0.976
N15—C201	1.4949 (10)	C29—C30	1.475 (8)
C16—C17	1.478 (8)	C29—H291	0.974
C16—H161	0.982	C29—H292	0.974
C16—H162	0.979	C30—C31	1.467 (9)
C17—C18	1.506 (9)	C30—H301	0.970
C17—H171	0.965	C30—H302	0.975
C17—H172	0.959	C31—H311	0.958
C18—C19	1.477 (9)	C31—H312	0.962
C18—H181	0.965	C31—H313	0.960
C18—H182	0.971	C251—H2511	0.961
C19—H191	0.960	C251—H2512	0.959
C19—H192	0.959	C251—H241	1.267
C19—H193	0.959	C251—H261	1.291
C20—C21	1.4948 (10)	C201—H2011	0.960
C20—C201	1.10 (3)	C201—H2012	0.960
C20—H201	0.952	C201—H244	1.238
C20—H202	0.956	H242—H244	0.681
			
Cl2—Sn1—C3	94.0 (2)	C25—C24—C251	55.8 (12)
Cl2—Sn1—C4	93.7 (2)	N15—C24—C201	59.9 (6)
C3—Sn1—C4	117.4 (4)	C25—C24—C201	168.3 (14)
Cl2—Sn1—C5	90.5 (2)	C251—C24—C201	135.4 (14)
C3—Sn1—C5	123.8 (4)	N15—C24—H241	145.6
C4—Sn1—C5	118.2 (4)	C25—C24—H241	100.2
Cl2—Sn1—O6	177.89 (12)	C251—C24—H241	55.5
C3—Sn1—O6	88.0 (2)	C201—C24—H241	87.3
C4—Sn1—O6	84.6 (3)	N15—C24—H242	118.1
C5—Sn1—O6	89.1 (3)	C25—C24—H242	87.9
Sn1—C3—H31	109.5	C251—C24—H242	97.3
Sn1—C3—H32	109.2	C201—C24—H242	87.0
H31—C3—H32	109.4	H241—C24—H242	66.0
Sn1—C3—H33	109.6	N15—C24—H243	102.1
H31—C3—H33	109.6	C25—C24—H243	54.8
H32—C3—H33	109.5	C251—C24—H243	97.4
Sn1—C4—H41	110.1	C201—C24—H243	115.2
Sn1—C4—H42	110.4	H241—C24—H243	101.5
H41—C4—H42	109.2	N15—C24—H244	97.5
Sn1—C4—H43	109.4	C25—C24—H244	124.3
H41—C4—H43	108.4	C251—C24—H244	117.2
H42—C4—H43	109.4	C201—C24—H244	51.1
Sn1—C5—H51	109.2	H241—C24—H244	65.1
Sn1—C5—H52	108.9	H242—C24—H243	44.3
H51—C5—H52	109.3	H242—C24—H244	36.4
Sn1—C5—H53	109.7	H243—C24—H244	74.9
H51—C5—H53	110.2	C24—C25—C26	115.5 (8)
H52—C5—H53	109.4	C24—C25—C251	62.1 (6)
Sn1—O6—S7	134.4 (2)	C26—C25—C251	62.1 (6)
O6—S7—O8	112.4 (3)	C24—C25—H251	107.2
O6—S7—O9	108.5 (3)	C26—C25—H251	126.4
O8—S7—O9	110.2 (3)	C251—C25—H251	118.7
O6—S7—O10	107.4 (2)	C24—C25—H252	120.1
O8—S7—O10	109.3 (3)	C26—C25—H252	83.8
O9—S7—O10	108.9 (3)	C251—C25—H252	137.2
Sn11^ii^—O9—S7	126.7 (2)	H251—C25—H252	101.8
S7—O10—Sn11	133.5 (3)	C24—C25—H243	46.8
O10—Sn11—O9^i^	176.23 (15)	C26—C25—H243	113.6
O10—Sn11—C12	93.2 (2)	C251—C25—H243	96.4
O9^i^—Sn11—C12	84.1 (2)	H251—C25—H243	119.1
O10—Sn11—C13	89.6 (2)	H252—C25—H243	73.3
O9^i^—Sn11—C13	94.0 (2)	C25—C26—C27	112.0 (9)
C12—Sn11—C13	121.2 (4)	C25—C26—C251	55.8 (12)
O10—Sn11—C14	85.3 (2)	C27—C26—C251	148.9 (19)
O9^i^—Sn11—C14	93.6 (2)	C25—C26—H261	108.5
C12—Sn11—C14	119.2 (4)	C27—C26—H261	108.6
C13—Sn11—C14	119.6 (4)	C251—C26—H261	58.8
Sn11—C12—H121	108.5	C25—C26—H262	109.2
Sn11—C12—H122	109.1	C27—C26—H262	109.1
H121—C12—H122	110.0	C251—C26—H262	102.0
Sn11—C12—H123	109.3	H261—C26—H262	109.5
H121—C12—H123	109.1	C25—C26—H263	148.8
H122—C12—H123	110.9	C27—C26—H263	98.9
Sn11—C13—H131	110.7	C251—C26—H263	99.0
Sn11—C13—H132	110.1	H261—C26—H263	63.1
H131—C13—H132	108.5	H262—C26—H263	53.9
Sn11—C13—H133	108.9	C25—C26—H264	62.9
H131—C13—H133	109.5	C27—C26—H264	98.8
H132—C13—H133	109.1	C251—C26—H264	98.9
Sn11—C14—H141	110.8	H261—C26—H264	152.3
Sn11—C14—H142	108.2	H262—C26—H264	55.7
H141—C14—H142	110.3	H263—C26—H264	109.5
Sn11—C14—H143	109.2	C26—C27—H271	109.8
H141—C14—H143	109.6	C26—C27—H272	108.9
H142—C14—H143	108.6	H271—C27—H272	109.1
C16—N15—C20	112.5 (8)	C26—C27—H273	110.5
C16—N15—C24	124.8 (8)	H271—C27—H273	109.6
C20—N15—C24	108.5 (7)	H272—C27—H273	109.0
C16—N15—C28	107.4 (6)	N15—C28—C29	114.9 (8)
C20—N15—C28	108.3 (7)	N15—C28—H281	110.3
C24—N15—C28	92.5 (8)	C29—C28—H281	108.8
C16—N15—C201	135.5 (12)	N15—C28—H282	106.8
C24—N15—C201	66.0 (11)	C29—C28—H282	106.2
C28—N15—C201	115.4 (14)	H281—C28—H282	109.6
N15—C16—C17	112.6 (8)	C28—C29—C30	118.1 (10)
N15—C16—H161	108.1	C28—C29—H291	106.7
C17—C16—H161	109.2	C30—C29—H291	105.9
N15—C16—H162	107.9	C28—C29—H292	108.0
C17—C16—H162	108.9	C30—C29—H292	107.6
H161—C16—H162	110.1	H291—C29—H292	110.4
C16—C17—C18	108.2 (8)	C29—C30—C31	116.5 (11)
C16—C17—H171	110.1	C29—C30—H301	107.9
C18—C17—H171	110.8	C31—C30—H301	106.2
C16—C17—H172	109.0	C29—C30—H302	107.7
C18—C17—H172	108.1	C31—C30—H302	108.6
H171—C17—H172	110.5	H301—C30—H302	110.0
C17—C18—C19	109.8 (13)	C30—C31—H311	109.4
C17—C18—H181	110.0	C30—C31—H312	109.5
C19—C18—H181	109.7	H311—C31—H312	109.8
C17—C18—H182	109.0	C30—C31—H313	109.3
C19—C18—H182	109.0	H311—C31—H313	109.2
H181—C18—H182	109.4	H312—C31—H313	109.6
C18—C19—H191	108.8	C24—C251—C26	115.5 (8)
C18—C19—H192	109.1	C24—C251—C25	62.1 (6)
H191—C19—H192	109.6	C26—C251—C25	62.1 (6)
C18—C19—H193	109.5	C24—C251—H2511	120.8
H191—C19—H193	109.7	C26—C251—H2511	121.7
H192—C19—H193	110.1	C25—C251—H2511	138.1
N15—C20—C21	124.6 (8)	C24—C251—H2512	102.6
N15—C20—C201	68.5 (7)	C26—C251—H2512	101.9
C21—C20—C201	68.5 (7)	C25—C251—H2512	142.1
N15—C20—H201	113.9	H2511—C251—H2512	79.8
C21—C20—H201	111.8	C24—C251—H241	48.0
C201—C20—H201	108.7	C26—C251—H241	116.6
N15—C20—H202	90.5	C25—C251—H241	99.4
C21—C20—H202	89.9	H2511—C251—H241	111.3
C201—C20—H202	128.2	H2512—C251—H241	54.8
H201—C20—H202	123.0	C24—C251—H261	154.5
C20—C21—C22	108.9 (8)	C26—C251—H261	39.0
C22—C21—C201	125.2 (17)	C25—C251—H261	96.6
C20—C21—H211	109.5	H2511—C251—H261	84.2
C22—C21—H211	116.8	H2512—C251—H261	85.8
C201—C21—H211	117.3	H241—C251—H261	131.5
C20—C21—H212	105.5	C24—C201—C21	154 (3)
C22—C21—H212	111.5	C24—C201—N15	54.1 (6)
C201—C21—H212	120.6	C21—C201—N15	124.7 (8)
H211—C21—H212	9.4	C24—C201—C20	122.0 (7)
C20—C21—H213	100.6	C21—C201—C20	68.5 (7)
C22—C21—H213	113.8	N15—C201—C20	68.5 (7)
C201—C21—H213	117.0	C24—C201—H2011	95.2
H211—C21—H213	12.3	C21—C201—H2011	63.6
H212—C21—H213	4.9	N15—C201—H2011	125.8
C20—C21—H214	105.4	C20—C201—H2011	127.6
C22—C21—H214	116.6	C24—C201—H2012	91.4
C201—C21—H214	116.4	C21—C201—H2012	103.5
H211—C21—H214	4.9	N15—C201—H2012	128.0
H212—C21—H214	5.8	C20—C201—H2012	120.6
H213—C21—H214	7.5	H2011—C201—H2012	90.7
C21—C22—C23	101.9 (13)	C24—C201—H244	46.1
C21—C22—H221	109.7	C21—C201—H244	111.1
C23—C22—H221	111.8	N15—C201—H244	88.8
C21—C22—H222	112.9	C20—C201—H244	147.6
C23—C22—H222	111.8	H2011—C201—H244	49.1
H221—C22—H222	108.7	H2012—C201—H244	91.4
C22—C23—H231	108.6	C24—H241—C251	76.5
C22—C23—H232	110.0	C24—H242—H244	86.7
H231—C23—H232	109.4	C24—H243—C25	78.4
C22—C23—H233	109.4	H242—H244—C24	56.9
H231—C23—H233	109.5	H242—H244—C201	138.4
H232—C23—H233	109.9	C24—H244—C201	82.8
N15—C24—C25	113.9 (8)	C26—H261—C251	82.2
N15—C24—C251	143.6 (15)		

Symmetry codes: (i) −*x*+1/2, *y*, *z*+1/2; (ii) −*x*+1/2, *y*, *z*−1/2.

### Hydrogen-bond geometry (Å, º)

**Table:**

*D*—H···*A*	*D*—H	H···*A*	*D*···*A*	*D*—H···*A*
C16—H161···O8^iii^	0.98	2.50	3.386 (18)	150 (1)
C12—H123···O6	0.96	2.46	3.311 (18)	148 (1)
C28—H281···O8^iii^	0.97	2.43	3.195 (18)	136 (1)
C25—H251···O8^iii^	0.95	2.48	3.434 (18)	175 (1)

Symmetry code: (iii) *x*, *y*+1/2, *z*−1/2.
